# RETRACTION: Research on the Realization Path of Fitness Strategy under the Guidance of the “Healthy China” Era

**DOI:** 10.1155/johe/9817895

**Published:** 2025-06-02

**Authors:** Journal of Healthcare Engineering

RETRACTION: B. Wang, “Research on the Realization Path of Fitness Strategy under the Guidance of the “Healthy China” Era,” *Journal of Healthcare Engineering* 2022, no. 1 (2022): 1–5, https://doi.org/10.1155/2022/2102076.

The above article, published online on 31 January 2022 in Wiley Online Library (https://wileyonlinelibrary.com), has been retracted by John Wiley & Sons Ltd.

This article has been retracted following an investigation undertaken by the publisher. This investigation has uncovered evidence of one or more of the following indicators of systematic manipulation of the publication process:

1. Discrepancies in scope
2. Discrepancies in the description of the research reported
3. Discrepancies between the availability of data and the research described
4. Inappropriate citations
5. Incoherent, meaningless, and/or irrelevant content included in the article
6. Manipulated or compromised peer review

The presence of these indicators undermines our confidence in the integrity of the article's content, and we cannot, therefore, vouch for its reliability. Please note that this notice is intended solely to alert readers that the content of this article is unreliable. We have not investigated whether authors were aware of or involved in the systematic manipulation of the publication process.

We regret that the usual quality checks did not identify these issues before publication and have since put additional measures in place to safeguard research integrity.

We wish to credit our own Research Integrity and Research Publishing teams and anonymous and named external researchers and research integrity experts for contributing to this investigation.

The authors were informed of our decision to retract the article but did not provide a response.

